# Oncolytic Effect of Zika Virus in Neuroendocrine Pancreatic Tumors: New Perspectives for Therapeutic Approaches

**DOI:** 10.3390/ijms242417271

**Published:** 2023-12-08

**Authors:** Martina Maria Cocco, Claudia Carcione, Vitale Miceli, Rosaria Tinnirello, Cinzia Maria Chinnici, Carmine Carbone, Giovanni Zito, Pier Giulio Conaldi, Gioacchin Iannolo

**Affiliations:** 1 Department of Research, IRCCS ISMETT (Istituto Mediterraneo per i Trapianti e Terapie ad alta Specializzazione), Via E. Tricomi 5, 90127 Palermo, Italy; mmcocco@ismett.edu (M.M.C.); vmiceli@ismett.edu (V.M.); rtinnirello@ismett.edu (R.T.); cchinnici@fondazionerimed.com (C.M.C.); gzito@ismett.edu (G.Z.); pgconaldi@ismett.edu (P.G.C.); 2 Fondazione Ri.MED, 90133 Palermo, Italy; ccarcione@fondazionerimed.com; 3 Medical Oncology, Fondazione Policlinico Universitario Agostino Gemelli, IRCCS, 00168 Rome, Italy; carmine.carbone@policlinicogemelli.it

**Keywords:** Zika virus, pancreatic cancer, pancreatic neuroendocrine neoplasm, glioblastoma, neuroendocrine tumors, pancreas, organ failure, transplantation

## Abstract

Pancreatic cancer (PCa) is the fifth leading cause of cancer mortality. Recently, our group and others have demonstrated the oncolytic activity of the Zika virus (ZIKV) against glioblastoma. The peculiar features of this virus offer the opportunity to use an agent already tested in vivo through natural transmission, with minimal effects on adults, to specifically target a tumor such as glioblastoma. This remarkable specificity prompted us to explore the potential use of ZIKV oncolytic action against other tumor types. In particular, we focused on the subgroup of pancreatic tumors with a neuroendocrine origin known as neuroendocrine tumors (NETs). We found that ZIKV exerts its oncolytic activity by specifically infecting NET cells, leading to growth inhibition and cell death. We also assessed whether the oncolytic action could be extended to pancreatic tumors different from NETs. However, as expected, the viral specificity is limited to NETs and is not applicable to adenocarcinoma tumors, indicating a narrow spectrum of action for this virus. These findings support the potential use of ZIKV in therapeutic approaches not only in glioblastoma, but also against other tumors, such as neuroendocrine pancreatic tumors.

## 1. Introduction

Pancreatic cancer ranks among the deadliest of cancers, largely due to its rapid progression and limited treatment options [1]. Neuroendocrine tumors (NETs), a less common type of pancreatic cancer, arise from hormone-producing cells [2]. Among them, pancreatic neuroendocrine neoplasm (pNEN), previously named “islet cell tumor”, is a fairly rare tumor, with an incidence of less than 1 case per 100,000 individuals per year. NETs can be classified into four categories according to the 2019 WHO classification [3]. This classification is determined by factors such as cancer cell appearance, growth rate (Ki-67 index), mitotic count, and the existence of necrosis. These categories include well-differentiated NETs (G1, G2, G3) and poorly differentiated neuroendocrine carcinomas (NECs). NECs account for around 10% to 20% of all neuroendocrine neoplasms (Table 1).

pNEN constitutes 7% of all NETs, with the lowest survival rate at 5 years among gastroenteropancreatic (GEP) NETs [4,5,6]. Neuroendocrine cells not residing in nervous system compartments display neural characteristics such as membrane excitability and/or hormone secretion [7]. The origin of these cells, ascribed to the neural crest, prompted us to investigate whether their neural properties can be effectively exploited to target NETs [8]. In this context, recent studies have shown that Zika virus (ZIKV) specifically targets neural stem cells (NSCs) and glioblastoma stem cells (GSCs) [9,10,11], exerting an oncolytic effect on glioblastoma in vitro and in vivo [10,11,12]. ZIKV belongs to the *Flaviviridae* family, and was originally isolated from a rhesus monkey in the Zika forest (Uganda, 1947) [13]. ZIKV transmission is driven by different species of Aedes mosquitoes. In adults, ZIKV infection is typically asymptomatic, causing symptoms (e.g., rash, mild fever, and joint pain that last for a few days) in less than 20% of cases [12,14,15]. In rare cases, ZIKV infection may lead to neurological disorders, such as Guillain–Barré syndrome (GBS) [12,16]. However, the most significant effect in humans is related to neurodevelopmental disorders. Notably, it has been demonstrated that ZIKV specifically targets NSCs [9]. This unique feature has prompted several groups to investigate ZIKV’s ability to inhibit GCSs and, consequently, to exert an oncolytic effect against glioblastoma. The effectiveness of ZIKV against glioblastoma has been clearly demonstrated in various laboratories using in vitro and in vivo models [12]. It has been shown that ZIKV enters NSCs through the neural cell adhesion molecule (NCAM1) receptor [17], suggesting that that specific neurotropism of ZIKV may be related to the expression of this receptor. Given ZIKV neural specificity and its limited adverse effects in adults, we sought to evaluate whether its oncolytic effect could be effectively used in the treatment of NETs.

## 2. Results

### 2.1. Cell Morphological Evaluation after ZIKV Infection

**Figure 1:**
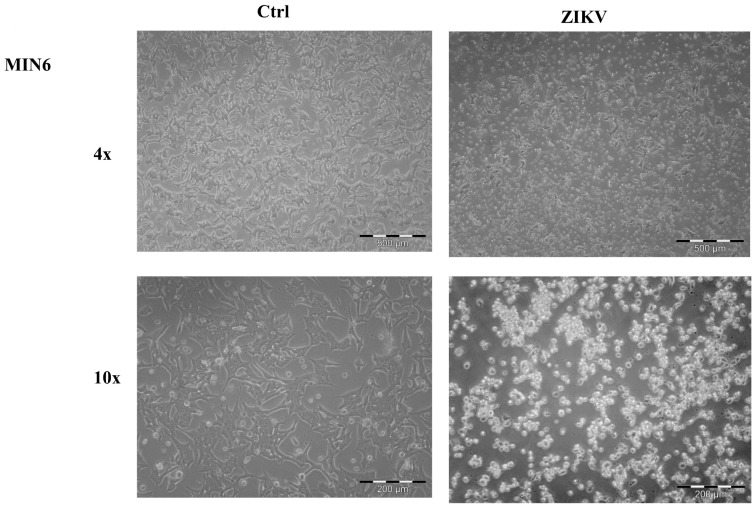
Morphological evaluation of MIN6 control (Ctrl) and after ZIKV infection (ZIKV). Images in bright field microscopy (4× and 10× magnification). After infection, the cell morphology appears clearly modified, and the cellular density is lower than control cells.

**Figure 2:**
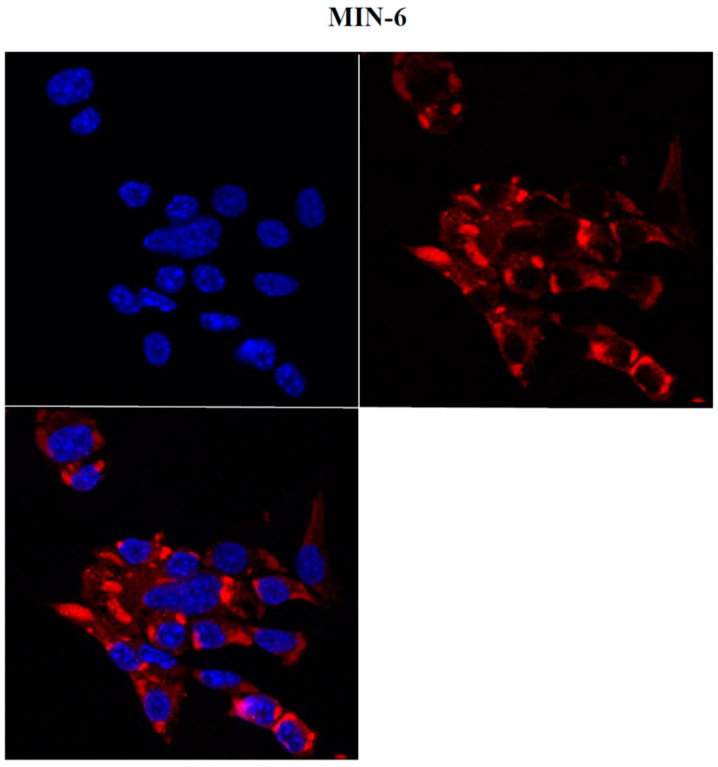
Evaluation of MIN6 ZIKV-infected cells by confocal microscopy (63×). Two days after infection, viral Zika glycoprotein E (red) showed cytoplasmic localization (nuclei in blue, DAPI).

ZIKV (ZIKV-strain Asian genotype-) was used to investigate its effect on the NET cell line, MIN6. ZIKV was produced as previously described [10], and viral supernatant was used to infect low-density growing MIN6. After one week, cells treated with ZIKV displayed a noticeable morphological change (Figure 1), suggesting a significant influence of the virus on these cells. To ensure specificity, we also evaluated whether other pancreatic tumors can be affected by ZIKV. In particular, we utilized two adenocarcinoma cell lines, ASPC1 and PANC1. No morphological changes were observed in either cell line following infection (Figure S1), suggesting that the effect of ZIKV may be specific to NET cells. To confirm whether the effect on MIN6 was not merely due to viral contact, as well as to establish viral active entry into the cells, we carried out an immunofluorescence analysis using a confocal microscope after ZIKV infection. Within a few days, the presence of viral glycoprotein E was detected in the cytoplasm of infected MIN6 cells (Figure 2).

**Figure 3:**
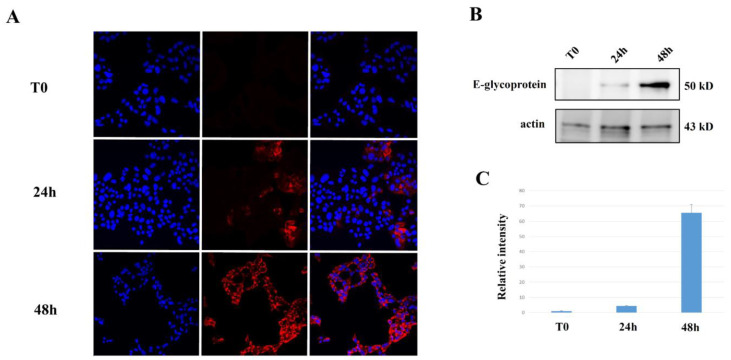
Time course of MIN6 infection with production of viral particles: MIN6 were infected with ZIKV and analyzed at T0, 24 h, and 48 h after infection by (**A**) confocal microscopy (63×). Viral Zika glycoprotein E (red) showed cytoplasmic distribution (nuclei in blue, DAPI). (**B**) Western blot analysis on MIN6 expression of glycoprotein E after ZIKV infection. (**C**) Relative intensity of normalized glycoprotein E vs. actin.

Time course analysis at 24 and 48 h showed a clear increase in the spreading of ZIKV after MIN6 infection, which correlates with E glycoprotein (Figure 3).

### 2.2. NCAM Expression in Pancreatic Tumor Cells

**Figure 4:**
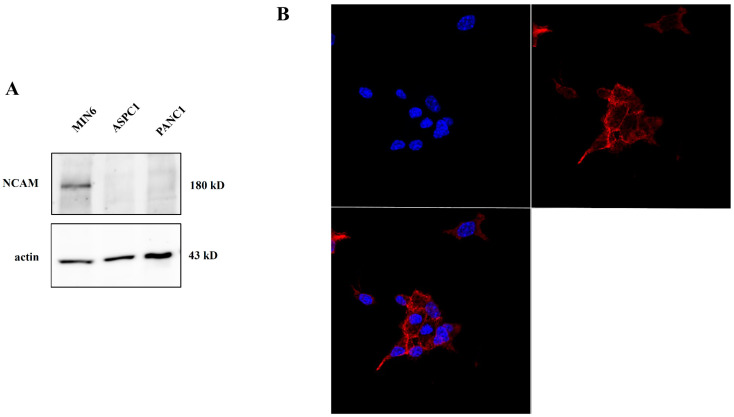
Evaluation of NCAM expression: (**A**) Western blot analysis on MIN6, ASPC1, and PANC1. NCAM expression is evident in MIN6 (NET model) and absent in the others (adenocarcinoma model). (**B**) Immunofluorescence analysis using confocal microscopy (63×) of NCAM (red) with clear staining, distributed on cell membrane (nuclei in blue, DAPI).

Given the role of the NCAM receptor in ZIKV entry into NSCs [17], we evaluated the expression of this receptor in the MIN6 cell line, used as an NET in vitro model. We performed Western blot and immunofluorescence analyses to assess NCAM expression. Western blot revealed a specific band corresponding to the 180 kD isoform of the NCAM protein, also showing exclusive expression on NET cell MIN6, whereas this receptor was absent in the two adenocarcinoma pancreatic tumor cell lines (ASPC1, PANC1) (Figure 4A). Furthermore, confocal microscope observation of MIN6 cells evidenced a membrane-specific pattern in NCAM staining (Figure 4B). All these results support the conclusion that ZIKV may specifically exert its effect on NET cells.

### 2.3. Evaluation of ZIKV Replication in NET Cells

**Figure 5:**
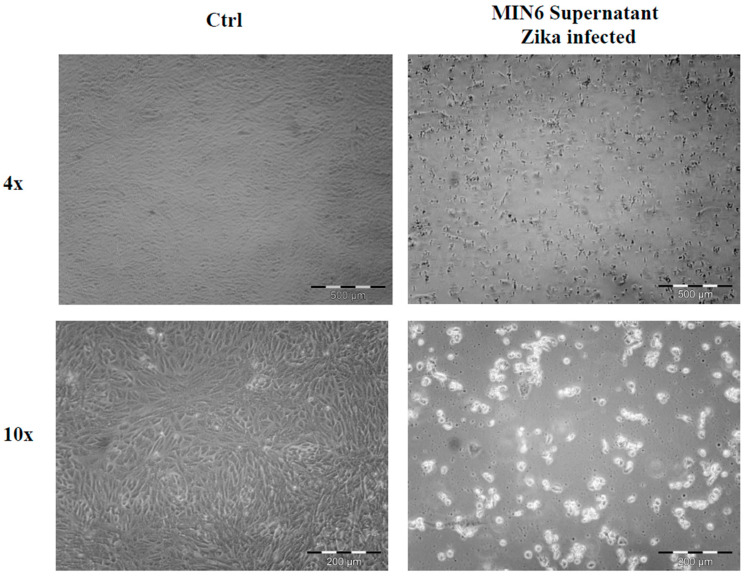
Evaluation of MIN6 ability to produce fully infective viral particles: ZIKV MIN6 supernatant was used to infect VERO cells; after one week, cells were examined in bright field microscopy (4× and 10× magnification), showing clearly viral activity compared to control cells (Ctrl).

**Figure 6:**
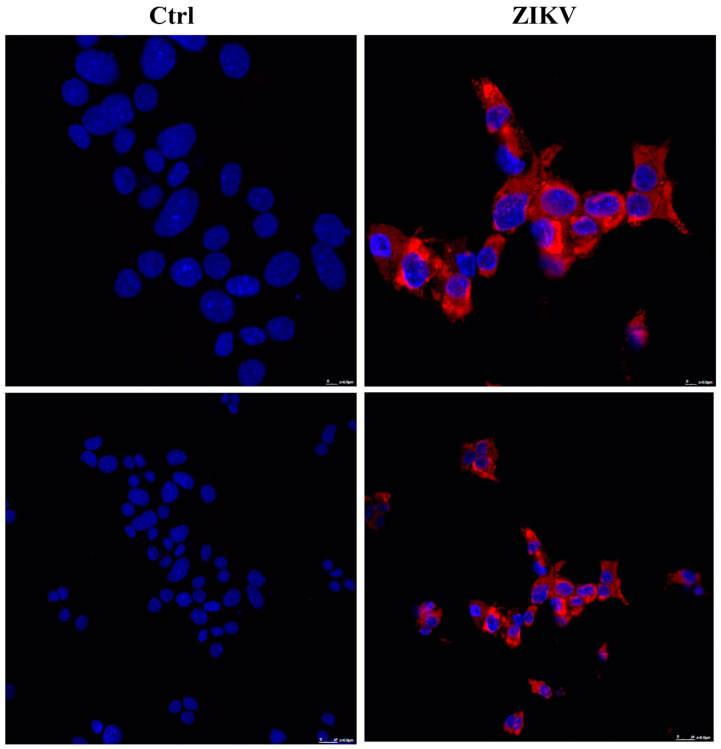
Evaluation of MIN6 ability to produce fully infective viral particles: MIN6 cells were reinfected with ZIKV and analyzed two days after reinfection by confocal microscopy (63×). Viral Zika glycoprotein E (red) showed cytoplasmic distribution (nuclei in blue, DAPI).

To evaluate the ability of ZIKV to actively replicate in NET cells, we collected the supernatant following MIN6 infection and quantified viral particles using specific RT-PCR with TaqMan assay, as previously described [10]. After one week, RT-PCR analysis on the supernatant from infected MIN6 revealed the presence of ZIKV particles in the order of 2.5 million/mL (+/−1.9%). Furthermore, we validated the infection potential of these particles in VERO cells by infecting them with the MIN6 ZIKV-derived supernatant (Figure 5). The viral replicative activity post reinfection was further confirmed by immunofluorescence on viral glycoprotein E (Figure 6), demonstrating that ZIKV replicates in MIN6, forming fully infective particles.

### 2.4. Evaluation of Cell Growth, Cell Cycle, and Cell Death after ZIKV Infection

**Figure 7:**
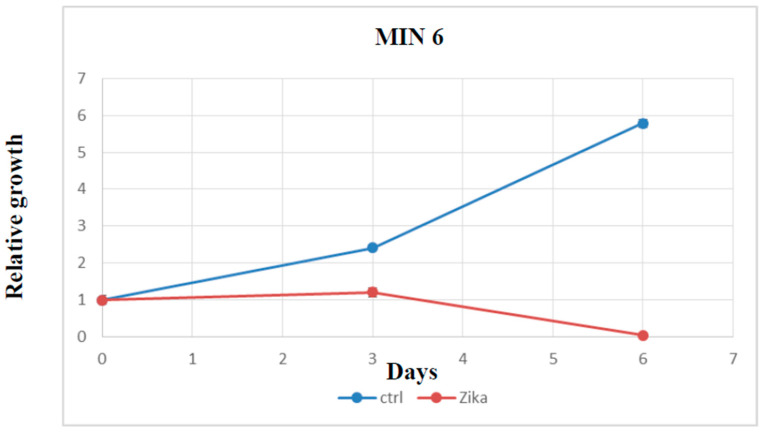
MIN6 growth curve after ZIKV infection vs. control (non-infected cells) evaluated by Cell Titer Glo (all reported experiments were carried out in triplicate (*p* value ≤ 0.05)).

**Figure 8:**
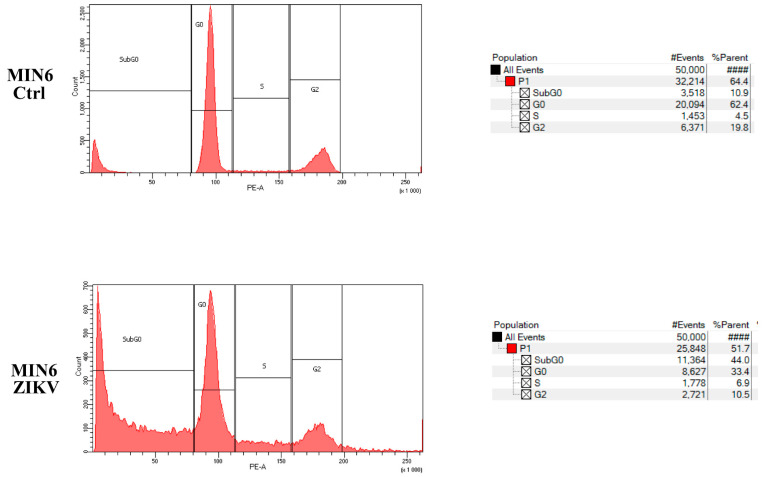
Propidium iodide cell cycle (PI) FACS analysis of Zika-infected MIN6 vs. control (one representative experiment is shown).

**Figure 9:**
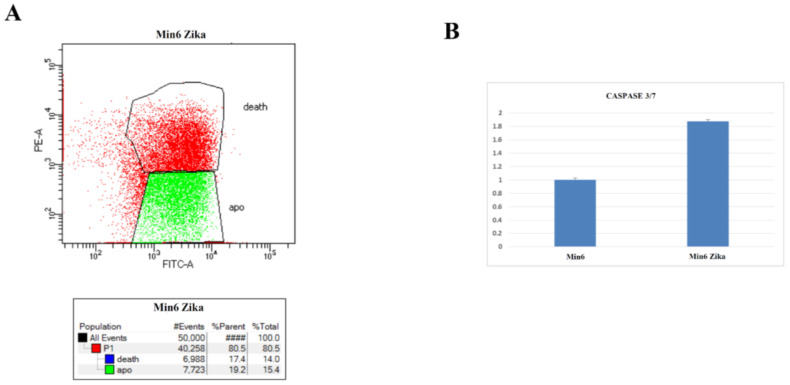
(**A**) Annexin/PI analysis of Zika-infected MIN6 (one representative experiment is shown). (**B**) Caspase3/7 activity evaluated by luminescence after ZIKV infection vs. control (*p* value ≤ 0.05).

**Figure 10:**
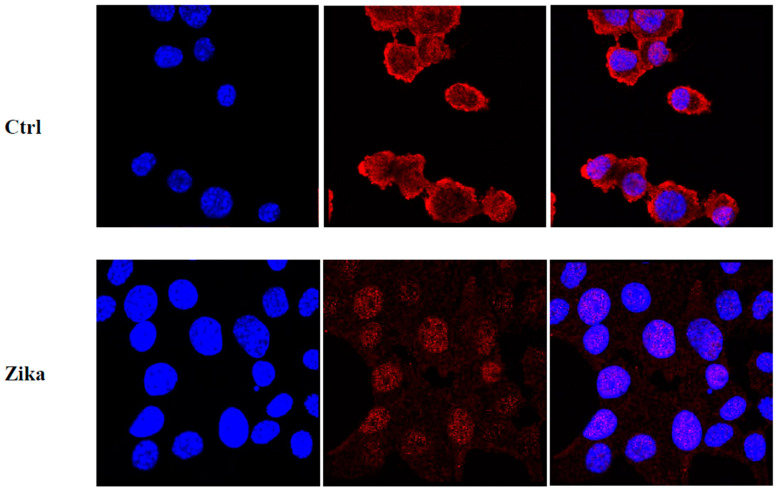
Bcl2 localization in MIN6 (control and Zika-infected): Bcl2 (red) showed cytoplasmic distribution in uninfected cells (nuclei in blue, DAPI) by confocal microscopy (63×), while Bcl2 was translocated into the nucleus after ZIKV infection.

To assess the in vitro effect of ZIKV on NET cells, we performed a cell growth assay after infection. Time course analysis on MIN6 clearly demonstrated the ability of ZIKV to induce cell growth reduction and promote cell death (Figure 7). Moreover, we evaluated whether ZIKV interferes with the cell cycle and/or induces cell death in MIN6. The assessment of DNA content using PI staining post infection revealed a dramatic increase in SubG0 fraction and a modification of the cell cycle profile in ZIKV-infected cells (Figure 8). Furthermore, we quantified the percentage of apoptotic induction by Annexin/PI analysis, demonstrating an increase in cell death, and apoptotic induction by caspase 3/7 activation in MIN6 ZIKV-infected cells (Figure 9). To gain insight into the mechanism of apoptotic induction after ZIKV infection, we performed an immunofluorescence analysis on Bcl2 (Figure 10). The staining revealed a clear translocation of Bcl2 into the nucleus. In this compartment, Bcl2 became an apoptotic inducer (Figure 10) [18,19].

To further confirm the effect of ZIKV infection on MIN6, we used the real-time growth system xCELLigence. The analysis revealed that after infection the cell growth undergoes a sudden arrest (Figure S2). The same analysis performed on PANC1 did not reveal any effect on growth (Figure S3) and there was no induction of a Sub G0 increase after infection (Figure S4).

## 3. Discussion

**Figure 11:**
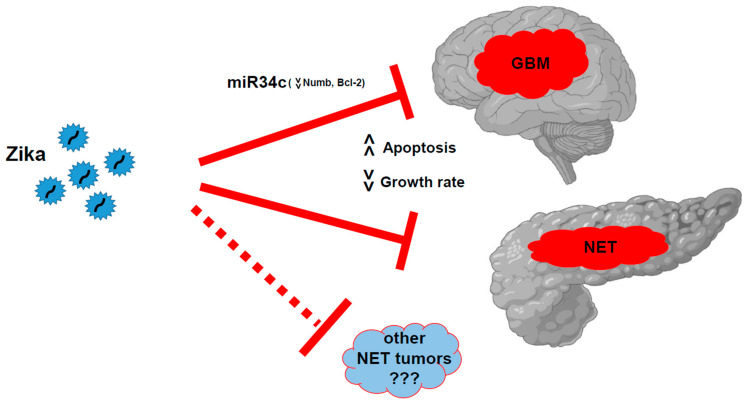
ZIKV oncolytic action vs. glioblastoma (inducing miR34c expression and Bcl2 reduction) and pancreatic NETs inducing cell death and growth arrest. Further analysis should be carried out to demonstrate whether this activity can be extended to other NETs.

Over the last 17 years, oncolytic viruses have been proposed as an innovative strategy for cancer treatment, following China’s authorization for oncolytic virus therapy [20]. Viruses such as herpes simplex virus 1 (HSV-1), adenovirus, vaccinia virus, reovirus, parvovirus, New Castle disease virus, and poliovirus have been used either modified or in their original forms [12]. However, despite the identification of ZIKV as a GBM oncolytic agent, to date no clinical trials have been undertaken to further investigate this. Except for a single reported case, no experimental protocols have been proposed in the last six years [21,22]. Nevertheless, ZIKV can be directly used in its natural form with minimal adverse effects in adults, and without widespread immunity in the population. In our previous study, we, along with other groups, demonstrated the remarkable oncolytic activity of ZIKV in GSCs [10,12]. Our strong interest in this virus is driven by its marked ability to specifically target glioblastoma cells, with no reported effect on other cell types, except on NSCs during the developmental stage [23]. In this regard, a case report described a possible fortuitous infection in a glioblastoma patient [24]. During a ZIKV outbreak in Brazil, the patient was subjected to neurosurgery after glioblastoma diagnosis. Twenty-four days after surgery, the patient showed seven days of symptoms compatible with ZIKV infection (fever, rash, arthralgia, and myalgia). Serum analysis found the presence of antibodies versus ZIKV. The patient experienced total remission, and the situation remained stable over the following six years [24]. In the present study, for the first time, we have demonstrated that the oncolytic effect of ZIKV could also target pancreatic NETs (Figure 11) due to their neural origin. We observed that MIN6 cells express the NCAM receptor, which may play a role in ZIKV-specific action. It remains to be clarified whether the presence of this receptor can be harnessed for the targeted treatment of other NETs. NCAM 180 kD isoform, which we identified in MIN6, has been described as the main target for polysialic acid (PSA) modification, and PSA NCAM is involved in cell adhesion, cell migration, and plasticity in the nervous system [25,26]. Notably, NCAM expression has been observed in various cancer types, including brain tumors, lung cancer, multiple myelomas, acute myeloid leukemia, and rhabdomyosarcoma [27]. Further investigations are needed to establish whether additional mechanisms are involved. For example, in GSCs, we previously observed that miR34c was involved in the induction of ZIKV oncolytic effects [10]. However, we did not observe any changes in miR34 family expression after ZIKV infection in MIN6 (data not shown). This suggests that the ZIKV oncolytic effect may depend on specific pathways that are not yet fully understood. Moreover, it has been proposed that neural stemness may play a role in tumorigenicity [28]. It is important to consider the differences in the differentiation of NE neoplasms (NENs), as the World Health Organization 2017 classification distinguishes well-differentiated neoplasms, such as NETs, and poorly differentiated neoplasms, such as neuroendocrine carcinomas (defined as NECs) [4].

However, ZIKV could well represent a unique tool for tumor targeting. Its effect on adult individuals has been previously assessed through standard infection, with minimal adverse outcomes. With our work, we have demonstrated that ZIKV oncolytic action is not limited to glioblastoma but can be applied to other tumors (Figure 11). Recently, a small trial investigated the effect of ZIKV in healthy humans to evaluate the effect of ZIKV in direct immunization (Evaluation of Two Zika Viruses for Use in Controlled Human Infection Models (CHIM) Clinicaltrial.gov NCT05123222) [29]. Nevertheless, at present, its potential role in cancer clinical trials or compassionate studies remains unexplored.

## 4. Materials and Methods

### 4.1. Cell Culture, Transfection, and Infection

Human pancreatic cancer cell lines ASPC1 and PANC1 were obtained from the American Type Culture Collection (ATCC, Manassas, VA, USA) and cultured as indicated by the provider [30]. Mouse insulinoma cells MIN6 were cultured and expanded as previously described [31]. ZIKV, strain H/PF/2013—Asian genotype 13 (provided by Dr. Giovanni Rezza, Department of Infectious Diseases, ISS Italian National Institute of Health, Rome under MTA) was propagated using VERO cells (ATCC) as previously described [10].

For the growth assays, cells were plated in 6–96 well plates (Corning, New York, NY, USA). At the indicated time points, the cells were collected and lysed using Cell Titer-Glo Luminescent Cell Viability Assay reagent (Promega, Mannheim, Germany). Caspase activity was evaluated using an Apotox Triplex Assay (Promega). Luminescence was measured using a Spark Microplate Reader (Tekan, Männedorf, Switzerland).

Cell growth was measured in real time using xCELLigence technology (ACEA, San Diego, CA, USA). The xCELLigence system was used according to manufacturer’s instructions. Briefly, after background impedance determination, MIN6 cells (2.5 × 10^4^ cells/well) were seeded into 16-well E-plates and incubated for 30 min at room temperature before being set in a Real-Time Cell Analyzer station in a conventional cell incubator at 37 °C and 5% CO_2_. After 21 h, cells were infected with ZIKV and analyzed for the indicated time. The impedance was measured every 15 min and expressed as cell index (CI), defined as (Rn − Rb)/15, where Rn is the impedance at a given time point and Rb is the background impedance. Slopes of growth curves were determined during the following 119 h after treatment.

### 4.2. RNA Extraction and RT-PCR

Zika virus RNA was purified from the cellular supernatant using QIAsymphony DSP Virus/Pathogen Midi Kit (937055, Qiagen, Germantown, MD, USA). Subsequently, the RNA was reverse transcribed and amplified with TaqMan-validated assays for Zika Virus (A31745, Applied Biosystems, Waltham, MA, USA). All analyses were carried out in triplicate. Standard quantified ZIKA control sample was obtained from European Virus Archive Global (EVA-GLOBAL, Marseille, France). Real-time data were analyzed using Microsoft Excel with the following formula: Expression level = 2^−ΔΔCt^ method. All experiments were conducted as independent triplicates and analyzed using standard deviation (SD). The *p*-value was calculated using Student’s *t*-test.

### 4.3. Immunofluorescence

Cell staining was conducted on cells seeded on multichamber slides (Nunclone, Sigma-Aldrich). Cells were washed in PIPES buffer (80 mM PIPES pH 6.8; 5 mM EGTA; 2 mM MgCl_2_, Sigma-Aldrich, St. Louis, MO, USA) and fixed with 4% paraformaldehyde/PIPES (Sigma-Aldrich) for 10 min. Subsequently, the cells were subjected to block-permeabilization in PBS containing 0.2% BSA (Sigma-Aldrich) and 0.1% Triton X-100 (Sigma-Aldrich) for 10 min, followed by DAPI staining (Sigma-Aldrich). The antibodies were used at the recommended dilutions provided by the suppliers: mouse anti-Flavivirus E glycoprotein (ab214333, 1/200, Abcam, Cambridge, UK), NCAM (GTX 133217, 1/100 GeneTex, MI, USA), and rabbit anti-Bcl2 (sc-492, 1/50, Santa Crutz, CA, USA).

All microscope images were acquired with a Nikon system TE2000-S microscope (Nikon Instruments, Amsterdam, The Netherlands), equipped with an Olympus LC20 Camera and Olympus soft imaging LCmicro software 5.2 (Olympus, Milan, Italy), or with a Leica confocal station (Leica SP5 confocal system) mounted on a Leica DM6000 inverted microscope, equipped with an Argon-ion laser and PMT detectors (Leica Microsystems, Wetzlar, Germany).

### 4.4. Immunoblotting

Cells were lysed with a buffer containing 1% Triton X-l00, 50 mM HEPES (pH 7.5), 150 mM NaC1, 10% glycerol, 1.5 mM MgCl_2_, 5 mM EGTA, and a cocktail of protease inhibitors (4 mM phenyl methylsulfonyl fluoride and 100 mg/mL aprotinin) and phosphatase inhibitors (10 mM sodium orthovanadate and 20 mM sodium pyrophosphate, Sigma-Aldrich). Cell lysates were then processed: for direct immunoblot analysis, we used 15–30 μg of total cellular proteins, resuspended in 25 μL of loading buffer, boiled for 5 min, and loaded onto SDS-PAGE for Western blot (WB). The antibodies for WB were used according to the manufacturer’s specifications: rabbit anti-NCAM (GTX 133217, 1/1000 GeneTex, MI, USA), mouse anti-Flavivirus E glycoprotein (ab214333, 1/200, Abcam, Cambridge, UK), and mouse anti-beta-actin (sc-81178, 1/1000, Santa Cruz Biotechnology, Dallas, TX, USA). WBs were acquired using a ChemiDoc MP Imaging System (Bio-Rad Laboratories Inc., Hercules, CA, USA).

### 4.5. Propidium Iodide Cell Cycle Analysis

Subconfluent cells were treated as described in the Figure legends. The cells were harvested, washed with PBS, fixed by adding cold ethanol dropwise (70% final concentration), incubated in ice for 30 min, rewashed in PBS 1% BSA, resuspended, and incubated for 60 min at RT (light protected) with a solution of propidium iodide and RNAse (50 μg/mL and 250 µg/mL, respectively). The DNA content and cell cycle profile were analyzed using a BD FACS Canto II cytofluorimeter (BD Biosciences, State College, PA, USA).

## Figures and Tables

**Table 1:** NET classification into four categories according to WHO 2019. This ranking is determined by factors such as cancer cell appearance, growth rate (Ki-67 index), mitotic count, and the presence of necrosis. These categories include well-differentiated NETs (G1, G2, G3) and poorly differentiated neuroendocrine carcinomas (NECs). The NECs account for around 10% to 20% of all neuroendocrine neoplasms.

Diagnosis	Grading	Mitotic Index (HPF) *	Ki-67 (%) ^†^	Morphology
NET	1	<2/10	≤3	Well differentiated
NET	2	2–20/10	3–20	Well differentiated
NET	3	>20/10	>20	Well differentiated
NEC - Small cell - Large cell	3	>20/10	>20	Poorly differentiated
MiNEN	-	-	-	Well—Poor differentiated
Tumor-like lesions	-	-	-	-

* 10 HPF = 2 mm^2^, at least 40 fields (at ×40 magnification) evaluated in areas of highest mitotic density. ^†^ MIB1 antibody; percentage of 500–2000 tumour cells in areas of highest nuclear labelling. HPF: high-power field; MiNEN: mixed neuroendocrine/non-endocrine neoplasm; NEC: neuroendocrine carcinoma; NET: neuroendocrine tumour; WHO: World Health Organization.

## Data Availability

The original contributions presented in the study are included in the article/Supplementary Materials; further inquiries can be directed to the corresponding author.

## Supplementary Materials

The following supporting information can be downloaded at: https://www.mdpi.com/article/10.3390/ijms242417271/s1.
