# Supplementary material for: Oncolytic Effect of Zika Virus in Neuroendocrine Pancreatic Tumors: New Perspectives for Therapeutic Approaches
Source: Int J Mol Sci. 2023 Dec 8;24(24):17271. doi: 10.3390/ijms242417271 (PMC10743494; doi:10.3390/ijms242417271)
Supplement: Supplementary file 1 [file ijms-24-17271-s001.zip › ijms-2726904-supplementary.pdf]

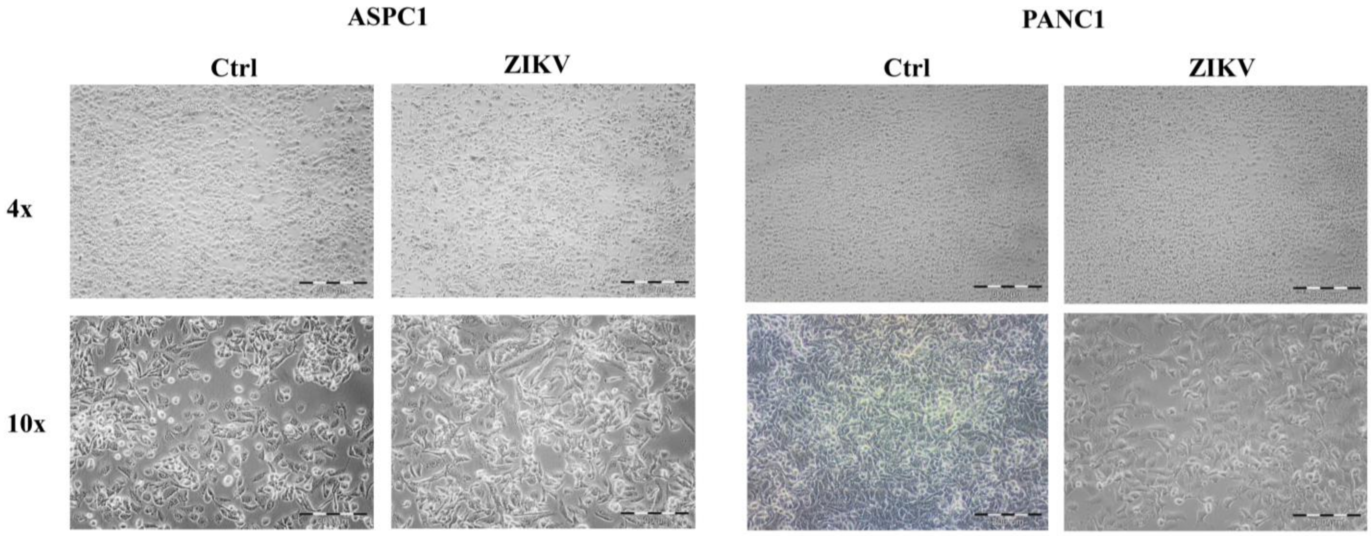

Figure S1. Morphological evaluation of ASPC1 and PANC1 control (Ctrl) and Zika-infected cells (ZIKV) in bright field microscopy (4X and 10X magnification). After infection, the cell morphology did not change, and the cellular density appeared uniform.

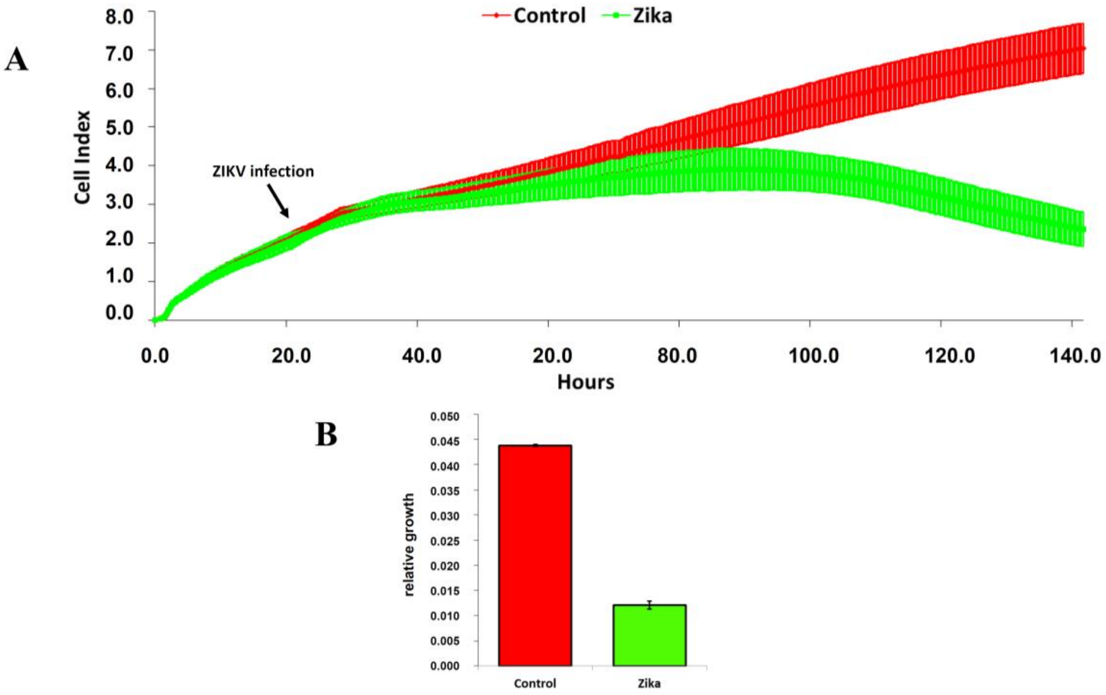

Figure S2. MIN6 growth curve obtainted by xCELLigence system. (A) 21 hours after plating the cells were infected (black arrow) with ZIKV (green) vs. control (non-infected cells, red). The impedance was measured every 15 minutes. (B) Growth curves were quantified during the following 119 hours after treatment through the following formula: relative growth = slope x time + intercept (p value ≤ 0.05).

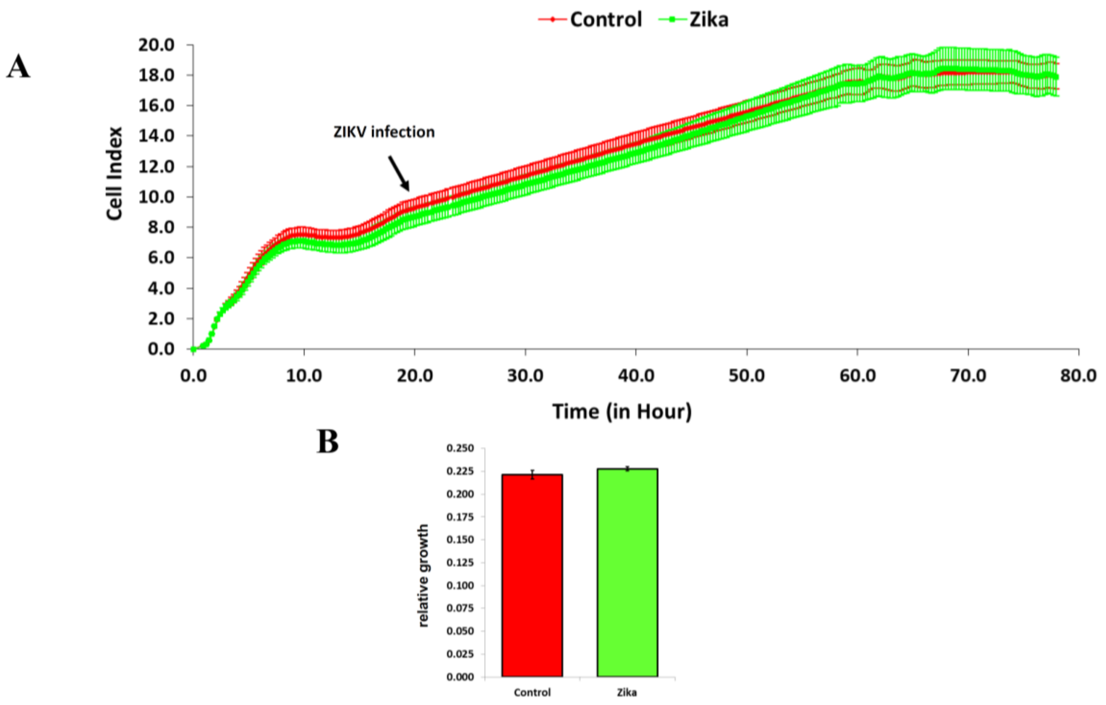

Figure S3. PANC1 growth curve obtainted by xCELLigence system. (A) 21 hours after plating the cells were infected (black arrow) with ZIKV (green) vs. control (non-infected cells, red). The impedance was measured every 15 minutes. (B) Growth curves were quantified during the following 80 hours after treatment through the following formula: relative growth = slope x time + intercept (p value ≤ 0.05).

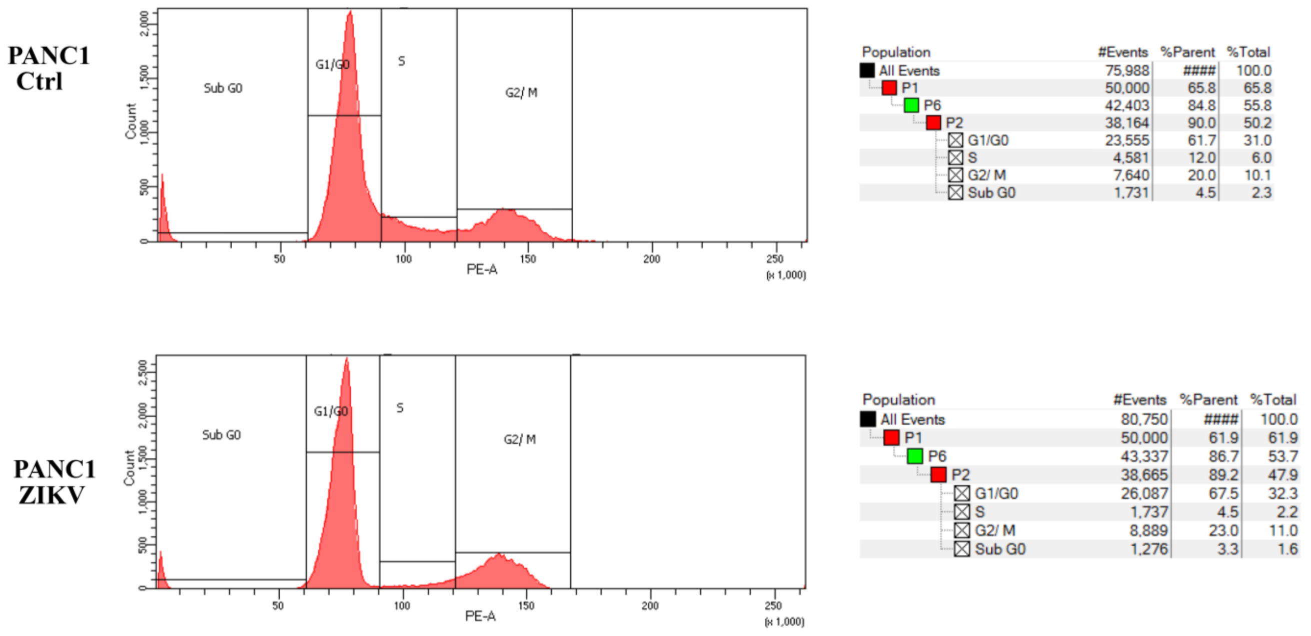

Figure S4. Propidium iodide cell cycle (PI) FACS analysis of Zika-infected PANC1 vs. control (one representative experiment is shown).
